# Evaluating the quality of peer interactions in children and adolescents with autism with the Penn Interactive Peer Play Scale (PIPPS)

**DOI:** 10.1186/s13229-017-0144-x

**Published:** 2017-06-17

**Authors:** Rebecca M. Jones, Andrew Pickles, Catherine Lord

**Affiliations:** 1 Center for Autism and the Developing Brain, Department of Psychiatry, Weill Cornell Medicine, 21 Bloomingdale Road, White Plains, NY 10605 USA; 2 Department of Biostatistics, Institute of Psychiatry, King’s College London, London, UK

**Keywords:** Autism spectrum disorder (ASD), Peer interactions, Penn Interactive Peer Play Scale (PIPPS), Teacher ratings

## Abstract

**Background:**

A core difficulty for individuals with autism is making friends and successfully engaging and interacting with peers. The majority of measures to assess peer interactions are observations in a school setting or self-report. The present study examined the convergent validity of using a teacher rating scale, the Penn Interactive Peer Play Scale (PIPPS), for collecting information about the quality of peer interactions at school.

**Methods:**

Teachers completed the PIPPS for 107 children with ASD when the child was 9 and 13 years of age. Clinicians completed diagnostic and cognitive assessments and caregivers completed the Autism Diagnostic Interview-Revised (ADI-R) when the child was 9.

**Results:**

Parent report of reciprocal friendships from the ADI-R was associated with teacher report about how socially connected the child was at school on the PIPPS, indicating strong convergence between teachers and parents. Children with more severe restricted and repetitive behaviors and lower verbal abilities were less connected with peers. Children with access to typical peers had more connections with peers compared to those who were in a special education classroom.

**Conclusions:**

The findings suggest that teacher ratings from the PIPPS can accurately capture the quality of peer interactions in children and adolescents with ASD and may be useful for clinicians and researchers to evaluate peer engagement in the classroom.

## Background

Autism spectrum disorder (ASD) is characterized by impairments in social communication skills including difficulties with forming and maintaining relationships [1]. A core difficulty is making friends and successfully engaging and interacting with peers. Elementary school children with ASD have fewer reciprocal friendships than typically developing classmates [2–6]. While it is well established that individuals with ASD have difficulties with peer relationships, there are few reliable methods for identifying difficulties with peer interactions in the classroom setting from teachers. The present study examined the convergent validity of using the Penn Interactive Peer Play Scale (PIPPS) for collecting teacher reports on the quality of peer interactions at school and the implications for friendships in children with ASD.

Accurately identifying the quality of peer relationships in children with ASD is complicated because self-report is more difficult to collect from children with ASD versus typically developing children. Previous research has used a variety of methods including parent report [7], observations in a school setting [8–10], and self-report by the child [5] as well as teacher report [4, 11] to capture the difficulties children with ASD experience in peer interactions and maintaining friendships. Teacher report is vital for accurately capturing peer interactions in the classroom. While there are a variety of scales commonly used to measure peer relationships in both typical and atypical development, very few include teacher report. One of the more commonly used in children with ASD is the Social Rating Scale System (SRSS) [12] a multi-informant questionnaire (teacher, parent, and self-report) that has two subscales (social skills and problem behaviors) that broadly measure pro-social behaviors, problem behaviors, and academic performance. The SRSS is often used as an outcome measure to assess changes in social functioning in response to treatments in children with autism [13, 14]. Other measures have relied on specific peer/friendship items from the Strengths and Difficulties Questionnaire completed by teachers [15] or The Pupil Evaluation Inventory—Teacher (PEI) [16] which measures global peer acceptance. Thus, prior research has queried teachers with measures that are fairly broad in terms of either social functioning or friendships.

The PIPPS is a teacher rating scale that specifically targets play skills and interactions with peers [17, 18]. The PIPPS was initially developed for preschool and kindergarten age children to understand social competence and its predictive value for academic success [19–21]. While the measure has largely been used in typically developing samples [22], some studies extended it to children who were maltreated [23] and a few studies used the PIPPS in young children with developmental delays including ASD [24, 25]. Unlike other social or friendship measures such as the SRSS, the PIPPS targets the quality and quantity of peer interaction. We used the PIPPS to measure peer interactions and compared it to parents’ reports of friendships to determine the accuracy of the teacher reports on the PIPPS.

Peer interactions in ASD are influenced by access to typically developing peers. Children with ASD who are exposed to typical peers in a school setting versus those who are only exposed to children with delays are described as having a higher quality of social interactions [26]. Research suggests that children with ASD who have typically developing friends engage in more sophisticated play and communication versus those who only have friends with ASD [27] (yet see [28] for a meta-analysis on friendships in ASD). Exposure to typical peers in a classroom may enhance social development and is a consideration when studying peer interactions in ASD. We recorded school placements with access to typically developing peers as well as the role of verbal abilities for all participants.

Children who have less severe ASD symptoms are expected to be more connected to their peers. Prior research has shown that children with lower overall ASD symptoms, as measured by the Autism Diagnostic Observation Schedule (ADOS), had higher social network salience, i.e., were more connected to other children in their classroom [29]. This is similar to the research finding that children with more severe autism traits as measured by the Social Responsiveness Scale (SRS) had more difficulties with peer relationships as rated by parents [30]. It is not known how the two domains of ASD symptoms (social communication and restricted and repetitive behaviors) may be differentially associated with the quality of peer interactions nor is it known how basic social communication (gestures, eye contact) versus impairments in interaction quality [31] may be related to peer interactions in ASD.

The goal of the present study was to examine the quality of peer interactions in children and adolescents and to determine the convergent validity of the PIPPS scale in evaluating classroom interactions in individuals with ASD. First, we examined the relationship between severity of ASD symptoms, verbal abilities, and quality of peer interactions. We predicted that children with less severe ASD social communication symptoms and higher verbal abilities, as assessed by a clinician, would have higher quality of peer interactions as rated by their teachers. Second, to further understand the quality of peer interactions in ASD, we compared children who had access to typically developing peers in their classroom versus those who did not and predicted that children with higher quality peer interactions would have access to typically developing peers. Last, to determine convergent validity, we compared teacher reports of peer interactions to parent reports of friendship on the Autism Diagnostic Interview-Revised (ADI-R) and predicted that there would be consistency among teacher and parent reports.

## Methods

### Participants

Participants were referrals of children under 37 months of age who were suspected of having possible autism or developmental delays. All children were from NC or metropolitan Chicago, IL. Seventy-five percent of the 213 original participants received ASD diagnoses at age 2 [32]. More detailed descriptions of the sample can be found elsewhere [33].

This study includes a subset of 107 children (94 males) out of the 213 initial participants who had a diagnosis of ASD at age 9 and also had at least one Penn Interactive Peer Play Scale (PIPPS) [17, 18] completed by their teacher. Of the 107 children, 72% were identified as Caucasian, 25% African American, 2% Asian, and 1% biracial. When the child was 9, 24% of the children’s mothers had completed a graduate degree, 38% a 4-year college degree, 22% some college or an associates’ degree, and 15% high school, and for 1 child, data was missing. Thirty-six percent of the children were in general education classrooms with access to typical peers, and 64% were in special education classrooms. Informed consent was obtained from all families. This research was approved by the appropriate IRBs.

### Autism diagnostic and cognitive testing

All children received a battery of ASD diagnostic and cognitive (IQ) testing. IQ tests were determined based on the developmental level of the child from the Mullen Early Scales of Learning (MSEL) [34], the Merrill-Palmer Scale of Mental Tests [35], the Differential Ability Scales (DAS)-Preschool [36], the DAS—School Age, the Raven’s Progressive Matrices [37], or the Wechsler Intelligence Scale for Children—III [38]. Some individuals had scores that fell within standardized norms; for those that did not, ratio IQs were calculated. These were calculated by dividing each individual’s “age equivalent” by the individual’s chronological age and multiplying by 100.

Autism diagnoses were based upon an in-person visit by a clinician who administered the ADI-R, a semi-structured interview between a trained clinician and caregiver [39], and the Autism Diagnostic Observation Schedule (ADOS) an observational measurement administered by a trained clinician [40]. Clinicians made a best estimate diagnosis based on all information. In order to compare autism severity across children with varying language abilities, calibrated severity scores (CSS) were generated from the ADOS [40]. The CSS is scored from 1 to 10 with 1 reflecting little to no symptoms and 10 reflecting severe symptoms. The CSS has a total score which demonstrates overall ASD symptoms, as well as social affect (SA) and restricted and repetitive behaviors (RRB) totals [41] (see Table 1). As a secondary analysis, to compute the subdimensions of social communication symptoms (basic social communication and interaction quality), raw ADOS scores were summed from the ADOS items identified in [31]. Scores for basic social communication ranged from 0 to 8 and for interaction quality from 0 to 6, with higher scores reflecting more impairment. Three children were missing the ADOS, and 2 children were missing the cognitive testing (see Table 1). For the secondary social communication subdimension analysis, 65 children were included because children with Module 1 ADOS were missing the requisite items to compute “interaction quality.” Children were only excluded from analyses when they were missing data.

**Table 1:** Participant demographics of means and (standard deviations)

*N* (males)	Calibrated severity score (CSS) Total	CSS social affect	CSS restricted and repetitive behaviors	Verbal ratio IQ	Nonverbal ratio IQ
104 (91 M)	7.6 (1.8)	7.6 (1.8)	7.5 (2.3)	49.1 (36.8) *N = 105*	61.4 (33.3) *N = 105*

Three individuals were missing ADOS scores and two individuals were missing cognitive testing

### Penn Interactive Peer Play Scale

The PIPPS is a brief teacher rating scale that measures play skills and interactions with peers [18]. PIPPS were collected from teachers at two time points, the first when the child was an average age of 9 years old (range 6–11 years; SD = 1 year) and the second when the child was an average age of 13 years old (range 10–15 years; SD = 1 year). The PIPPS is a 32-item Likert-scale questionnaire that provides information about peer play behaviors in the classroom and at school. Teachers indicated how often they observed each behavior in the last 2 months, i.e., “never,” “seldom,” “often,” or “always.” There are three subscales of the PIPPS: (1) Play Interaction, which indicates the child’s play strengths and includes behaviors such as comforting, helping other children, showing creativity in play, and encouraging others to join play; (2) Play Disruption, which describes aggressive, antisocial behaviors that interfere with ongoing play interactions; and (3) Play Disconnection, which reflects withdrawn behavior and non-participation in peer play. Parents were asked to request that the teacher who knew the child best completed the PIPPS.

A number of PIPPS questionnaire records were incomplete. When 28 or more of the 32 items were present, the missing items were completed by prorating (assigning to missing items the subject specific mean of the remaining items that formed a subscale). For the age 9 data, items 8 and 32 were missing from all of the PIPPS questionnaires. In order to fill in these items, the regression coefficients for these items and the subscale scores (omitting items 8 and 32) were estimated from the age 13 data, and these coefficients and the corresponding age 9 subscale scores were used to predict the two missing items scores at age 9. After this prorating and imputation, 107 participants were missing four or fewer items and were included in all analyses; 65 children had data from age 9 or 13, and 42 children had data from both time points. Statistical analyses took into account repeated measures from the same child, and these details are outlined in sections below.

Raw totals from the three PIPPS subscales were converted to standard *T* scores, which were based on a mean of 50 and a standard deviation of 10. *T* scores were generated for the three subscales; there is no total score. *T* scores had been created from norms with children younger than the current participants (kindergarten age), so while we report *T* scores for easier interpretation and standardization with prior research, we also ran all analyses with raw scores. Slightly weakened effects between raw scores and *T* scores occurred in one case and are noted in results; all other findings remained significant.

### Parent ratings of friendship

To test the convergent validity of teacher reports from the PIPPS, we compared PIPPS scores to parent reports of their children’s friendships. The ADI-R is a semi-structured interview between a clinician and caregiver; questions target specific ASD-related behaviors. Questions are scored from 0 to 3 (similar to ADOS scoring conventions). There is a question on the ADI-R asking caregivers to report about their child’s reciprocal friendships and the quality of these friendships (the “Friendship” item). Because each individual in this sample has multiple ADI-R data points, we chose the friendship item from the ADI-R at age 9, as it was closest in time to when the PIPPS were collected. The “friendship” item has four possible ratings based upon the caregiver’s answers: 0 reflects a clear reciprocal friendship, 1 corresponds to a limited reciprocal friendship, 2 corresponds to contact with peers only in group settings, and 3 corresponds to no peer relationships. As used in previous research [42], we used the four scores on the friendship item as a categorical variable to reflect parent reports of friendship. Six participants were missing data from the ADI-R and were not included in this analysis.

### Statistical analyses

To initially determine whether there was a difference in PIPPS scores from age 9 versus age 13, we completed three independent sample *t* tests with the PIPPS subscales, and considered *p*s <0.017 significant.

Ultimately to increase power, PIPPS scores from ages 9 and 13 were analyzed together, using a linear mixed regression model estimated by maximum likelihood with a random intercept to account for the correlation of responses from the same participant. In order to determine whether there was an effect of time point (age 9 versus age 13), an additional interaction term of time point and prognostic factor was then tested to assess whether there was any unexpected prediction that was specific to one of the time points (age 9 or 13) (all such terms proved to be non-significant). All analyses described below were conducted with each subscale of the PIPPS separately as the dependent variable, and all analyses included a covariate of the age of the child at the time when the PIPPS was completed to control for the fact that the PIPPS were collected at a range of ages.

First, to better understand the quality of peer interactions in ASD, we wanted to determine the relationship between severity of ASD symptoms and verbal abilities with peer interactions. Using a linear mixed model as explained in the paragraph above, we included the calibrated severity score for social affect (CSS SA) and calibrated severity score for restricted and repetitive behaviors (CSS RRB) together as covariates (independent variables) with each PIPPS subscale *t* score separately as the dependent variable. The goal was to determine whether more severe social communication or RRB symptoms impacted peer interactions. We included the basic social communication and interaction quality subdimensions together as covariates in a distinct analysis as the independent variables to determine whether specific aspects of social communication impairments affected peer interactions. Separately, we included VIQ as a covariate to determine whether verbal abilities at the time the PIPPS was collected affected the quality of peer interactions.

Second, to determine convergent validity of whether teacher-reported peer interactions on the PIPPS were consistent with parent reports of their child’s peer interactions, we directly compared the PIPPS subscale scores to parent reports of friendships on the ADI-R. Scores from the friendship item on the ADI-R were included as a factor (independent variable) with each PIPPS subscale as the dependent variable in a linear mixed model.

The final set of analyses determined whether access to typically developing peers in a classroom influenced PIPPS scores, in order to better understand factors that may influence the quality of peer interactions in ASD. A binary variable of regular classroom (access to typically developing peers) versus special education classroom (no typically developing peers in the classroom) was included as a factor (independent variable) with each PIPPS subscale as the dependent variable. Given that children with higher verbal abilities are more often placed in mainstream classroom settings, we performed an analysis with VIQ as a covariate with the PIPPS subscales that demonstrated a significant relationship with classroom placement.

All results were considered significant at *p* < 0.05 unless otherwise stated. We followed up significant main effects from the linear mixed effects regression model with pairwise comparisons. For these pairwise comparisons, we report mean differences, standard error, and *p* values adjusted using Bonferroni correction. All analyses were conducted in SPSS version 24.

## Results

### Peer interactions by age

**Fig. 1:**
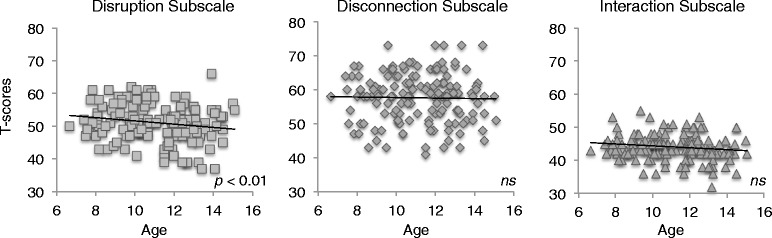
PIPPS subscale scores by age

There was a decline with age in how disruptive children were during play from age 9 to age 13 (*t*(147) = −2.7, *p* = 0.009) demonstrating that children with ASD were less aggressive and disruptive during peer interactions as they transitioned from childhood to early adolescence (see Fig. 1). In contrast, there was no significant difference between age 9 and age 13 for the interaction or disconnection subscales (*p'﻿*s >0.2). See Table 2 for descriptive statistics for each PIPPS subscale *T* score at age 9 and age 13. Higher scores on the interaction subscale reflect higher quality of peer interactions, whereas higher scores on the disruption and disconnection subscales reflect more aggression and less connection with peers respectively.

**Table 2:** Distribution of PIPPS subscale *T* scores divided by age groups

		Penn Interactive Peer Play Scale *T* scores		
	*N*	Interaction	Disruption	Disconnection
Age 9	72	44.46 (3.72) (36–55)	52.29 (5.29) (41–62)	57.53 (6.74) (43–73)
Age 13	77	43.66 (3.86) (32–53)	49.86 (5.82) (37–66)	57.68 (7.59) (41–73)

Means (standard deviations) (range of scores)

### Autism symptoms, cognitive abilities, and peer interactions

We assessed multiple factors that could influence peer interactions in ASD and found that children with more severe restricted and repetitive behaviors had less connection with peers (*F*(1,86.9) = 4.0, *p* = 0.048). Overall, social affect symptoms were not associated with peer connections (*p* = 0.089) nor were there associations with basic social communication (*p* = 0.339) or interaction quality (*p* = 0.716). Basic social communication (*p* = 0.074), interaction quality (*p* = 0.099) as well as the overall social affect and restricted and repetitive behaviors were not associated with the disruption subscale (*p'*s >0.11). There was no association between severity of autism symptoms and the interaction subscale (*p'*s >0.15).

As expected, children with higher verbal abilities were more connected with peers (*F*(1,91.2) = 12.5, *p* < 0.001) and also had higher levels of interactions during play (*F*(1,80.42) = 6.2, *p* = 0.015). The relationship between VIQ and amount of connection with peers remained significant even when controlling for the child’s classroom placement. Verbal abilities were not associated with the disruption subscale (*p* > 0.3). Raw PIPPS scores demonstrated a non-significant effect with the disconnection subscale and restricted and repetitive behaviors (*p* = 0.064).

### Parent and teacher report of peer interactions

**Fig. 2:**
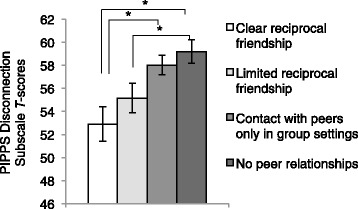
Convergence between teacher-rated PIPPS disconnection scores and parent ratings of friendship on the ADI-R

To determine convergent validity, we compared parent report of reciprocal friendships from the ADI-R at age 9 with teacher reports on the PIPPS and found that the ADI-R was associated with teacher reports about how socially connected the child was at school on the PIPPS at 9 and/or 13 (*F*(3,85.2) = 4.5, *p* = 0.006). These findings suggest strong convergence regarding the quality of peer interactions between teachers and parents (see Fig. 2). Pairwise comparisons demonstrated that children with clear reciprocal friendships, as reported by parents, were more connected to their peers (disconnection mean = 52.8) (lower scores reflecting greater connection with peers), as reported by teachers, compared to those whose parents reported that the child only had contact with peers in group settings (disconnection mean = 58.6) (mean diff = −5.9, SE = 2.0, *p* = 0.029) and to those whose parents reported that they had no peer relationships (disconnection mean = 59.5) (mean diff = −6.8, SE = 2.1, *p* = 0.01). There were no significant differences between children with some reciprocal friendships, as reported by parents compared to those whose parents had reported no peer relationships (*p* = 0.198). Parent reports of reciprocal friendships did not correspond to teacher ratings on the interaction or disruption subscales of the PIPPS (*p'*s *>*0.3).

### Age 9 classroom placement

**Fig. 3:**
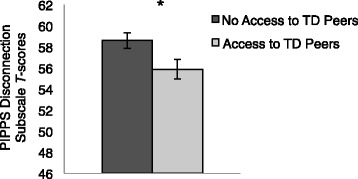
Children with access to typically developing (TD) peers had lower PIPPS disconnection scores compared to children who did not have access to TD peers

As expected, secondary analyses demonstrated that children in a classroom with typical peers compared to those in a special education classroom were rated by their teachers as having greater connections with their peers (*F*(1, 98.2) = 4.8, *p* = 0.031) (﻿see Fig. 3). There were no significant relationships between the interaction or disruption subscales and classroom placement (*p*s >0.7). Also as expected, children in a classroom with typical peers had higher verbal IQs (VIQs) (M = 83; SD = 34) and nonverbal IQs (NVIQs) (M = 92; SD = 24) at age 9 compared to those in a special education classroom (VIQ M = 30; SD = 21; NVIQ M = 44; SD = 24). The relationship between connection with peers and classroom placement was no longer significant when VIQ was included as a covariate, thus the relationship could be fully explained by VIQ (*F*(1, 87.8) = 6.6, *p* = 0.012).

## Discussion

The current study describes teacher ratings of peer interactions on the PIPPS in children and adolescents with ASD. Parent reports of friendships converged with teacher reports of peer interactions. Children with more severe restricted and repetitive behaviors and lower verbal abilities as measured by a clinician were less connected with peers as rated by teachers. In addition, children with access to typical peers had more connections with peers compared to those who were in a special education classroom, with the caveat that those with access to typical peers also had higher cognitive abilities. Together, the findings suggest that the PIPPS can capture the quality of peer interactions in children and adolescents with ASD and may be a useful tool for clinicians and researchers who are interested in assessing peer engagement in the classroom.

Convergence between teacher reports of peer interactions and parent reports of friendship was good. Consistency in reporting between parent and teachers is often rare in children with ASD, particularly for social behaviors [43–45]. Comparisons of reporting between parents and clinicians are also often inconsistent [46]. It is likely that children with ASD exhibit different types of social behaviors at school when interacting with peers as compared to at home when social interactions are often primarily with siblings or other family members. The consistency between the ADI-R friendship item and the PIPPS teacher scale suggests that the PIPPS accurately reflects the difficulties of children with ASD in engaging with their peers. The PIPPS has a parent version. Future research that compares PIPPS scores on the parent version versus the teacher version could provide more definitive insight into stability between parent and teacher reports of peer interactions as reported by the PIPPS.

Children who had access to typically developing peers had a higher quality of peer interactions compared to those who were in special education classrooms. Our findings support a growing body of literature highlighting the importance of exposure to typical peers for children with ASD, at least those with high verbal skills, in promoting more sophisticated social interactions. However, a limitation is that, with this sample, it was impossible to disentangle whether children with overall better outcomes are initially given better opportunities and placed in regular classrooms. Thus, future research is needed to explore the link between access to typical peers at different time points during development (i.e., childhood versus adolescence); thus, it can help us to further understand different factors that influence successful peer relationships in ASD [29].

Children with less severe restricted and repetitive behaviors were more connected with peers. These findings are consistent with research suggesting that less severe restricted and repetitive behaviors early in life predict very positive young adult outcomes [47]. Difficulties in the restricted and repetitive behavior domain may be particularly challenging in terms of developing and maintaining peer relationships. There was no significant relationship between PIPPS scores with overall social communication symptoms as measured by the ADOS CSS Social Affect score and with the subdimensions of social communication [31]. The ADOS, while designed to assess social communication abilities, does not capture quality or quantity of interactions with same age peers. Thus, these findings are a reminder that an ADOS with a clinician may not provide a complete clinical picture of the social interaction difficulties inherent to ASD across multiple contexts.

The PIPPS disconnection subscale, unlike the interaction or disruption subscale, was related to ASD symptoms, parent reports of friendship, and whether a child had access to typically developing peers. The disconnection subscale items are well aligned with ASD symptoms, as they specifically ask teachers to rate how often the child: “withdraws,” “wanders aimlessly,” “is ignored by others,” and “needs help to start playing.” The friendship item on the ADI-R specifically targets the quality and quantity of same age peer interactions, thus the disconnection subscale questions likely align most closely to this question. It is possible that the disconnection subscale versus the interaction and disruption scales may be most useful for understanding social impairments in children with ASD. Future research comparing scores on the PIPPS in children with ASD versus typically developing children will be important to determine whether differences between diagnostic groups are present in all subscales, or specifically the disconnection subscale of the PIPPS.

### Limitations

A limitation to the present study is that the PIPPS was not normed for 9 or 13 year olds though the majority of analyses of raw data suggested use of standardized scores was reasonable and interpretable. Second, children’s placements in a classroom with typical peers versus more restricted special education classrooms were not based upon random assignment, an important distinction for interpreting results. Third, the current sample was impaired in terms of cognitive abilities. Thus, the PIPPS may be less suitable for older and more cognitively able children and adolescents with ASD, future research should test a diverse sample of ASD children to determine the broad utility of the measure. Last, the identity of the teachers completing the PIPPS was unknown, which means that we cannot be certain whether the same teacher filled out the PIPPS for the same child twice, although generally the teachers varied from year to year for each child.

### Future directions

The present study begins to address variability of peer relationships during early adolescence in ASD, but there is much still that we do not know. Future research in children with ASD that compares the PIPPS to other teacher report measures of peer interactions such as the SRSS can provide further evidence of the validity of the PIPPS for accurately capturing the difficulties inherent to children with ASD in this domain. It will also be important for future work to address what social or cognitive factors early in life predict successful peer interactions during early adolescence [7] and whether the quality of peer interactions during this time period subsequently relates to outcomes during young adulthood. Ultimately, information about the social trajectories and their relationship to peer interactions in ASD will help inform clinical treatment targets at different time points during development.

## Conclusions

The PIPPS teacher report is a brief questionnaire that captured peer interactions in children and young adolescents with ASD and converged with parent reports of friendship. Ultimately, the findings have implications for peer play in ASD as the PIPPS may be useful for clinicians and researchers who are interested in collecting information about relationships in the classroom to better understand social interaction impairments inherent to ASD.

## Abbreviations

ADI-R: Autism diagnostic interview – revised
ADOS: Autism diagnostic observation schedule
ASD: Autism spectrum disorder
CSS RRB: ADOS calibrated severity score restricted and repetitive behaviors
CSS SA: ADOS calibrated severity score social affect
NVIQ: Nonverbal IQ
PIPPS: Penn Interactive Peer Play Scale
VIQ: Verbal IQ
